# Clinical Outcome in Elderly Patients (Aged ≥ 65 Years) Treated with Chemotherapy for Advanced Soft Tissue Sarcomas: A Tokai Musculoskeletal Oncology Consortium Study

**DOI:** 10.3390/cancers18111849

**Published:** 2026-06-04

**Authors:** Tomoki Nakamura, Satoshi Tsukushi, Akihito Nagano, Tomohisa Sakai, Hisaki Aiba, Junji Wasa, Kozo Hosono, Yoji Shido, Yuya Izubuchi, Tetsuo Shimoyama, Katsuhisa Kawanami, Eiji Kozawa, Masahiro Hasegawa, Yoshihiro Nishida

**Affiliations:** 1 Department of Orthopaedic Surgery, Mie University Graduate School of Medicine, Tsu 514-8507, Japan; 2 Department of Orthopaedic Surgery, Aichi Cancer Center Hospital, Nagoya 464-8681, Japan; 3 Department of Orthopaedic Surgery, Gifu University Graduate School of Medicine, Gifu 501-1194, Japan; 4 Department of Orthopaedic Surgery, Nagoya University Graduate School of Medicine, Nagoya 466-8560, Japan; 5 Department of Orthopaedic Surgery, Nagoya City University Graduate School of Medicine, Nagoya 467-8602, Japan; 6 Division of Orthopaedic Oncology, Shizuoka Cancer Center Hospital, Shizuoka 411-8777, Japan; 7 Department of Orthopedic Oncology, Okazaki City Hospital, Okazaki 444-8553, Japan; 8 Department of Orthopaedic Surgery, Hamamatsu University School of Medicine, Hamamatsu 431-3192, Japan; 9 Department of Orthopaedics and Rehabilitation Medicine, Faculty of Medical Sciences, University of Fukui, Fukui 910-1193, Japan; 10 Department of Orthopaedic Surgery, Fujita Health University, Toyoake 470-1192, Japan; 11 Department of Orthopaedic Surgery, Aichi Medical University School of Medicine, Nagakute 480-1195, Japan; 12 Department of Orthopaedic Surgery, Nagoya Memorial Hospital, Nagoya 468-8520, Japan; 13 Department of Rehabilitation, Nagoya University Hospital, Nagoya 466-8560, Japan

**Keywords:** elderly, soft tissue sarcoma, chemotherapy, advanced stage

## Abstract

**Background/Objectives**: Chemotherapy is recommended for patients with advanced soft tissue sarcoma (STS). However, chemotherapy is less aggressive in elderly patients than in younger patients owing to comorbidities and other health problems. This multicenter study aimed to examine the outcomes of elderly patients treated with chemotherapy for advanced STS. **Methods**: The study cohort included 60 men and 71 women with a mean age of 73 years. The mean follow-up duration was 22.9 months. **Results**: As first-line treatment, the doxorubicin-containing regimen was the most frequently used. Dose reduction was more frequent in patients aged ≥ 75 years than in those aged ≤ 74 years. Complete response occurred in two patients and partial response in eight patients. The objective response rate was 8.2%. The 1- and 2-year survival rates after first-line chemotherapy were 61.8% and 40.2%, respectively. The median survival time was 19 months. In multivariate analysis, patients with performance status (PS) 2 or 3 had poorer survival than those with PS 0 or 1. The median survival times for patients with PS 0 or 1 and PS 2 or 3 were 22.1 and 4.3 months, respectively. Among 131 patients, no fatal adverse events occurred, although chemotherapy was discontinued due to adverse events in 28 patients. **Conclusions**: Chemotherapy for advanced STS in elderly patients may be effective in those with good PS, although it should be considered to evaluate the benefits and risks of cytotoxic chemotherapy.

## Simple Summary

This multicenter study aimed to examine the outcomes of elderly patients treated with chemotherapy for advanced soft tissue sarcoma. The study cohort included 131 patients with a mean age of 73 years. Patients with performance status (PS) 2 or 3 had poorer survival than those with PS 0 or 1 in multivariate analysis. Among 131 patients, no fatal adverse events occurred, although chemotherapy was discontinued due to adverse events in 28 patients. Chemotherapy for advanced STS in elderly patients may be effective in those with good PS, although it should be considered to evaluate the benefits and risks of cytotoxic chemotherapy.

## 1. Introduction

Globally, the number of elderly individuals is rapidly increasing. In 2026, 36.5%, 32.3%, 26.1%, and 24.9% of Japanese, German, UK, and US citizens, respectively, will be aged > 60 y, according to a World Health Organization report [1]. An aging population may increase demand for healthcare and long-term care services. Elderly people often face multiple health issues that may affect cancer treatment strategies. According to the Bone and Soft Tissue Tumor Registry in Japan, the incidence of soft tissue sarcoma (STS) is highest between the ages of 60 and 80 [2]. Of all patients diagnosed with STS, 20–50% present with clinically detectable metastases [3,4,5]. Chemotherapy is recommended for patients with advanced STS [6]. However, chemotherapy is less aggressive in elderly patients than in younger patients owing to comorbidities and other health problems [7,8]. In some cases, elderly patients or their relatives refuse chemotherapy. In other cases, the physician’s decision was based on the patient’s activities of daily living, performance status (PS), and comorbidities. These decisions may reflect evidence that these therapeutic options do not substantially improve prognosis. However, prolonged patient survival may be expected in the era of newer agents, such as pazopanib, trabectedin, and eribulin. This multicenter study aimed to examine the outcomes of elderly patients treated with chemotherapy for advanced STS in Japan since 2012, following the introduction of these agents.

## 2. Materials and Methods

Based on the findings of clinical trials in Japan, pazopanib (since 2012), trabectedin (since 2015), and eribulin (since 2016) can be administered regardless of prior doxorubicin-based chemotherapy or histological subtype. Therefore, we included Japanese patients from 12 hospitals who initiated chemotherapy for advanced STS between November 2012 and December 2023. Advanced STS was defined as unresectable local recurrence and distant metastasis.

The study cohort included 60 men and 71 women with a mean age of 73 y (range, 65–90 y) at the time of receiving first-line chemotherapy for advanced STS. The mean follow-up duration was 22.9 months (range, 1.1–103.3 months). Doxorubicin alone or doxorubicin and ifosfamide (AI) was administered to 28 patients as neoadjuvant and adjuvant chemotherapy.

The standard chemotherapy doses were as follows: Doxorubicin alone 60 mg/m^2^ or 75 mg/m^2^ (the 75 mg/m^2^ dose required institutional review board approval); doxorubicin (60 mg/m^2^) and ifosfamide (10 g/m^2^), eribulin (1.4 mg/m^2^), gemcitabine (900 mg/m^2^) and docetaxel (70 mg/m^2^) (GD), pazopanib (800 mg/day), trabectedin (1.2 mg/m^2^), paclitaxel (100 mg/m^2^).

The primary purpose of this study was to examine the prognostic factors associated with post-metastatic overall survival (OS) in elderly patients with STS who underwent chemotherapy for advanced STS. The secondary purpose was to determine whether treatment strategy and response to chemotherapy differed according to histological diagnosis.

### Statistical Analysis

Clinicopathological factors were evaluated using the Mann–Whitney U test (quantitative data) and the chi-square test (qualitative data). Tumor response to chemotherapy was evaluated according to the new response evaluation criteria in solid tumors (RECIST version 1.1). OS was defined as the period from the initial administration of first-line chemotherapy for advanced STS to death or the last follow-up. Survival curves were constructed using the Kaplan–Meier method. Univariate and multivariate analyses were conducted using the log-rank test and Cox proportional hazards model, respectively. Variables with *p* < 0.05 in univariate analysis were included in the multivariate model. All statistical analyses were performed using the EZR graphical user interface (Saitama Medical Center, Jichi Medical University, Saitama, Japan) for R Ver 1.70 (R Foundation for Statistical Computing, Vienna, Austria), a modified version of R Commander with functions commonly used in biostatistics. *p* values < 0.05 were considered statistically significant.

## 3. Results

### 3.1. Patient’s Characteristics

In total, 131 patients were included in this study (Table 1). The PS scores were 0 in 63 patients, 1 in 53, 2 in 12, and 3 in 3. Patients aged ≥ 75 y had higher PS than those aged ≤ 74 y (*p* = 0.0145, Mann–Whitney U test). As few patients had a PS of 3 (*n* = 3), PS 2 and 3 were combined into a single category (PS 2+). Primary tumor sites included the retroperitoneum (*n* = 37), thighs (*n* = 30), legs (*n* = 9), buttocks (*n* = 9), chest wall (*n* = 8), legs (*n* = 8), knees (*n* = 7), upper arm (*n* = 6), back (*n* = 6), and other sites (*n* = 11). STS histological subtypes were 33 dedifferentiated liposarcomas (DDLPS), 24 undifferentiated pleomorphic sarcomas (UPS), 20 leiomyosarcomas (LMS), 13 myxofibrosarcomas (MFS), 8 myxoid liposarcomas (MLPS), 6 malignant peripheral nerve sheath tumors (MPNST), and 27 tumors of other types.

Overall, 64 patients had unresectable local STS, 84 had lung metastases, 11 had liver metastases, 9 had bone metastases, and 18 had metastases to other sites. Specifically, 24 had unresectable STS alone, 69 had distant metastasis alone, and 38 had both unresectable STS and metastasis.

### 3.2. Tumor Response

As first-line treatment, the doxorubicin-containing regimen (*n* = 61) was the most frequently used, followed by eribulin (*n* = 33), pazopanib (*n* = 17), trabectedin (*n* = 7), and GD (*n* = 5) (Table 1). Initial dose reduction was performed in 69 patients (52.7%). Dose reduction was more frequent in patients aged ≥ 75 y (28/41, 68.3%) than in those aged ≤ 74 y (41/90, 45.6%) (*p* = 0.023, chi-square test). Among 131 patients, 122 had an evaluable tumor response. According to RECIST criteria, Complete response (CR) occurred in 2 patients (1.6%) with LMS and angiosarcoma, partial response (PR) in 8 patients (6.6%). The objective response rate was 8.2% (10/122). Age (65–74 vs. ≥75) was not associated with tumor response.

Table 2 shows the relationship between major chemotherapy agents and tumor response. CR occurred in one patient with LMS who received doxorubicin alone. PR occurred in three patients who received doxorubicin alone and five patients who received eribulin. CR occurred in another patient with angiosarcoma who received paclitaxel.

Table 3 shows the relationship between the regimen and histological diagnosis. Eribulin was frequently used in patients with DDLPS, whereas doxorubicin alone or AI was commonly administered to patients with other STS types as first-line treatment.

Three patients continued first-line chemotherapy at the final follow-up visit. Among those who discontinued first-line treatment, 86 patients (65.6%) received second-line therapy. As second-line treatment, eribulin (*n* = 27) was most frequently used, followed by pazopanib (*n* = 25), GD (*n* = 13), trabectedin (*n* = 9), doxorubicin (*n* = 8), and other drugs (*n* = 4). Of the 86 patients, 41 received third-line treatment. Eribulin (*n* = 15) was most frequently prescribed, followed by trabectedin (*n* = 12), pazopanib (*n* = 6), GD (*n* = 5), and other drugs (*n* = 3). Of the 41 patients, 17 received fourth-line treatment. Eribulin (*n* = 6) was the most frequently prescribed, followed by pazopanib (*n* = 4), trabectedin (*n* = 3), GD (*n* = 3), and other drugs (*n* = 1).

### 3.3. Survival

At final follow-up, 26 patients were alive with disease, and 105 had died. The 1- and 2-year survival rates after first-line chemotherapy were 61.8% and 40.2%, respectively. The median survival time was 19 months. Table 3 shows the median survival time according to major histological diagnosis. Patients with LMS and MLPS had longer median survival times, whereas those with UPS, MFS, and MPNST had median survival of <10 months (Table 4).

**Figure 1:**
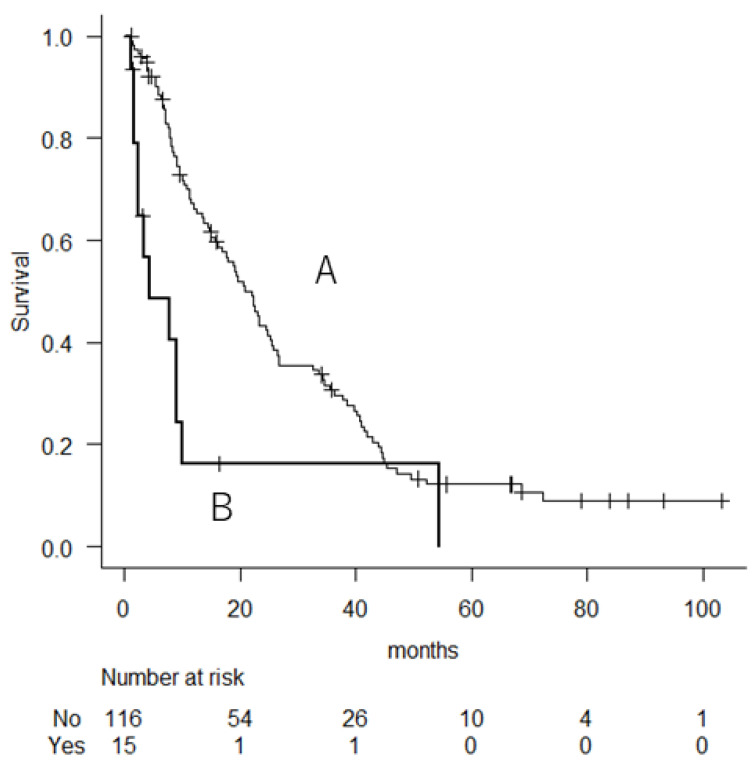
Kaplan-Meier curve shows disease-specific survival after first-line chemotherapy depending on performance status (A: PS 0 or 1, B: PS 2 or 3).

In all 131 patients, PS, tumor response to first-line therapy, and chemotherapy target were related to prognosis in the univariate Cox hazard proportional model analysis (Table 5). In multivariate analysis, patients with PS 2+ had poorer OS than those with PS 0 or 1 (HR 2.243, 95% Confidence Interval (CI) 1.109–4.539, *p* = 0.02467). The median survival times for patients with PS 0 or 1 and PS 2+ were 22.1 and 4.3 months, respectively (*p* < 0.0001, log-rank test) (Figure 1). There was no significant difference in survival between patients aged ≥ 75 y and those aged ≤ 74 y. Median survival was 22.5 months in 90 patients aged ≤ 74 y and 12 months in 41 patients aged ≥ 75 (*p* = 0.242, log-rank test). Similarly, survival did not differ between patients who received doxorubicin as first-line treatment and the other patients. Median survival time was 16.9 months in 70 patients treated with doxorubicin alone or AI and 22.3 months in 61 patients treated with other agents (*p* = 0.457, log-rank test). First-line chemotherapy agents and histological diagnosis were not associated with predicting survival.

### 3.4. Safety

Among 131 patients, no fatal adverse events occurred. Chemotherapy was discontinued due to adverse events in 28 patients (21.4%). Discontinuation rates due to adverse events were as follows: pazopanib (6/17; 35.3%), trabectedin (2/7; 28.6%), eribulin (8/33; 24.2%), AI (1/6; 16.7%), and doxorubicin (7/52; 13.5%). Reasons for discontinuation included pneumonia (*n* = 5), pneumothorax (*n* = 3), general fatigue (*n* = 3), liver disorders (*n* = 3), sepsis (*n* = 2), heart failure (*n* = 2), febrile neutropenia (*n* = 2), and other causes (*n* = 8).

## 4. Discussion

Among 131 elderly patients with advanced STS at 12 hospitals, the 1- and 2-year survival rates after first-line chemotherapy for advanced STS were 61.8% and 40.2%, respectively. The median survival time was 19 months. PS was a prognostic variable for predicting OS in multivariate analysis. A good PS is widely recognized as an independent predictor of survival in several varieties of cancer and sarcoma [9,10,11,12,13,14]. A doxorubicin-containing regimen (46.6%) was the most frequently used first-line therapy. Eribulin was frequently administered to patients with DDLPS. CR and PR were achieved in 10 of 122 patients (8.2%). Age (65–74 vs. ≥75) was not associated with OS or tumor response. Chemotherapy was discontinued due to adverse events in 28 patients (21.4%).

These patients were highly selected elderly individuals. Savina et al. reported that age ≥ 75 y was significantly associated with a lower probability of receiving systemic treatment [8]. In their analysis of 2165 patients with metastatic STS, 1429 of 1886 patients aged ≤ 74 y (75.8%) received chemotherapy, compared with 146 of 279 patients aged ≥ 75 y (52.3%). Overall, age was identified as a prognostic variable for advanced STS [15,16]. Karavasillis et al. reported a relative risk of death was 1.46 (95% CI: 1.10–1.92) in patients aged > 60 y compared with that in patients aged < 40 y [16]. However, we also emphasize that it is important not to deny life-prolonging treatment for elderly patients based exclusively on their chronological age.

Doxorubicin is the standard first-line chemotherapy for advanced STS [6,17], although Pautier et al. recently reported that induction therapy with doxorubicin and trabectedin, followed by trabectedin maintenance, was associated with improved overall and progression-free survival compared with doxorubicin alone in metastatic or unresectable uterine or soft-tissue LMS [18]. In this study, the median survival time after the administration of first-line chemotherapy was 19 months. Younger reported the outcomes of 348 elderly patients with advanced STS treated with first-line chemotherapy [17]. The median overall survival among those treated with doxorubicin was 9.8 months, and outcomes in elderly patients were only slightly worse than in younger patients. Nakamura et al. reported that the median post-metastatic survival of the 32 patients with musculoskeletal sarcoma who received no treatment for their metastasis was 7.2 months [19]. Therefore, chemotherapy for advanced STS may be considered in elderly patients, although we did not perform a comparison of OS between patients who received chemotherapy and those who did not receive it for advanced STS directly in this study.

In this study, eribulin was frequently administered to patients with DDLPS, possibly reflecting favorable outcomes in advanced liposarcoma treated with eribulin as first- or second-line chemotherapy [20,21,22,23,24]. Schöffski P et al. reported that patients with LMS and liposarcoma (LPS) in the eribulin group had significantly longer overall survival than those treated with dacarbazine (HR 0.77; 95% CI 0.62–0.95; *p* = 0.0169) in a phase 3 study [14]. Furthermore, Demetri et al. demonstrated improved progression-free survival with eribulin versus dacarbazine in a subgroup analysis of patients with liposarcoma (2.9 vs. 1.7 months) [25]. Nakamura et al. also reported a significant difference in progression-free survival (PFS) between patients with and without L-sarcoma treated with eribulin [22]. These findings support the hypothesis that eribulin is effective in L-sarcoma, particularly liposarcoma.

Savina suggested that the best supportive care should be considered after failure of second-line therapy [8]. However, their study included patients treated between 1990 and 2013. In this study, 41 patients (31.3%) received third-line treatment. In the era of newer agents such as pazopanib, trabectedin, and eribulin, further-line therapy may be considered if PS is maintained. Interestingly, Ikeda et al. reported the usefulness of chemotherapy for patients with poor PS non-small cell lung cancer by the inverse probability weighting (IPW) method, although commonly, patients with not only elderly but also poor PS are often considered to be particularly fragile and unfit for cytotoxic chemotherapy [23]. They compared the overall survival of the chemotherapy and best supportive care (BSC). In both PS2 and PS3 cohorts, IPW-adjusted overall survival was significantly longer in the chemotherapy group than in the BSC group (HR 0.42; 95% CI 0.32–0.55; *p* < 0.001 and HR 0.56; 95% CI 0.41–0.75; *p* < 0.003, respectively). They emphasized cancer cachexia, which was defined as weight loss > 5% over the past 6 months, was related to shorter overall survival, which was consistent with previous studies [23,24,25,26].

The American Society of Clinical Oncology recommends the use of geriatric assessment (GA) to evaluate the benefits and risks of cytotoxic chemotherapy in elderly patients [27]. GA-based tools, such as the Geriatric-8 or Vulnerable Elders Survey-13, are useful for estimating the risk of chemotherapy toxicity [27,28,29,30,31,32]. Chen et al. performed a meta-analysis including 42 studies with 9053 patients, and they reported the results from subgroup analyses, which showed that high G-8 scores were associated with increased OS (HR 1.97; 95% CI; 1.59–2.44; *p* < 0.001) and PFS (HR 1.73; 95% CI 1.43–2.10; *p* < 0.001) in patients treated with chemotherapy [28]. Lee et al. reported the association of the G-8 with treatment intensity and prognosis in 451 elderly patients with diffuse large B-cell lymphoma. They showed that the factors associated with the administration of the standard regimens, the G-8, remained as a significant factor in the multivariate logistic regression analyses, although standard therapy significantly contributed to a decreased mortality risk for patients with all G-8 scores [31]. Therefore, future clinical trials are needed for elderly patients with advanced STS incorporating GA, given the global aging population, for the association of the G-8 with treatment intensity and prognosis. In this study, chemotherapy was discontinued due to adverse events in 28 patients (21.4%), although most had a PS of 0 or 1 and more than half underwent dose reduction at initial administration. The pre-treatment assessment using the GA-based tool should be administered.

This study was retrospective, and comparison with previous phase 3 trials may be limited due to differences in tumor subtypes, eligibility, and follow-up procedures. The elderly patients were highly selected. We also should understand the survival results in this study with explicit caution due to the histological subtypes with small sizes.

## 5. Conclusions

Chemotherapy for advanced STS in elderly patients may be effective in those with good PS, although it should be considered to evaluate the benefits and risks of cytotoxic chemotherapy.

## Figures and Tables

**Table 1:** Patient’s background.

Patient Characteristics	*n*
Age	
65–74	90
≥75	41
Sex	
Men	60
Women	71
Performance status	
0	63
1	53
2+	15
Treatment	
Doxorubicin	55
Doxorubicin and ifosfamide	6
Eribulin	33
Pazopanib	17
Trabectedin	7
Gemcitabine and docetaxel	5
Paclitaxel	4
Others	4
Full dose intensity of initial administration	
Yes	62
No	69
Histological cell type	
Dedifferentiated liposarcoma	33
Undifferentiated pleomorphic sarcoma	24
Leiomyosarcoma	20
Myxofibrosarcoma	13
Myxoid liposarcoma	8
Malignant peripheral nerve sheath tumor	6
Fibrosarcoma	4
Angiosarcoma	4
Synovial sarcoma	4
Others	15
Neoadjuvant chemotherapy at primary STS	
Yes	32
No	99
Site of disease involvement	
primary site involved	64
lung metastasis	84
liver metastasis	11
bone metastasis	9
other metastases	18

STS: soft tissue sarcoma, PS2+: PS2 and 3.

**Table 2:** The relationship between major agents and tumor response.

Agent	Tumor Response				
	CR	PR	SD	PD	NE
A or AI (*n* = 61)	1	3	30	26	1
Eriburin (*n* = 33)	0	5	17	6	5
Pazopanib (*n* = 17)	0	0	9	6	2
Trabectedin (*n* = 7)	0	0	4	3	0

A: Doxorubicin, AI: Doxorubicin-ifosfamide, CR: Complete response, PR: Partial response, SD: Stable disease, PD: Progressive disease, NE: not evaluated.

**Table 3:** The relationship between histological diagnosis and regimen.

Histology	*n*	Median Survival Time (95% CI)
DDLPS	33	16 months (8.5–22.3)
UPS	24	9 months (5.8–17.8)
LMS	20	34.2 months (15.7–42)
MFS	13	9.5 months (5.9–23.3)
MPLS	8	34.2 months (5.3–NA)
MPNST	6	8.9 months (2.4–NA)
Others	27	25.2 months (9.5–33.5)

DDLPS: De-differentiated liposarcoma, UPS: Undifferentiated pleomorphic sarcoma, LMS: Leiomyosarcoma, MFS: Myxofibrosarcoma, MLPS: Myxoid liposarcoma, MPNST: Malignant peripheral nerve sheath tumor, ( ): complete response or partial response, 95% CI: 95% confidence interval.

**Table 4:** Median overall survival according to histological diagnosis.

Histology	*n*	Median Survival Time (95% CI)
DDLPS	33	16 months (8.5–22.3)
UPS	24	9 months (5.8–17.8)
LMS	20	34.2 months (15.7–42)
MFS	13	9.5 months (5.9–23.3)
MPLS	8	34.2 months (5.3–NA)
MPNST	6	8.9 months (2.4–NA)
Others	27	25.2 months (9.5–33.5)

DDLPS: De-differentiated liposarcoma, UPS: Undifferentiated pleomorphic sarcoma, LMS: Leiomyosarcoma, MFS: Myxofibrosarcoma, MLPS: Myxoid liposarcoma, MPNST: Malignant peripheral nerve sheath tumor, ( ): complete response or partial response, 95% CI: 95% confidence interval.

**Table 5:** Cox proportional analysis for predicting survival.

		Univariate Analysis			Multivariate Analysis		
Variables		HR	95% CI	*p* Value	HR	95% CI	*p* Value
Age	Years	1.017	0.979–1.056	0.397			
Age	65–74	1					
	≥75	1.288	0.842–1.972	0.244			
Sex	Women	1			1		
	Men	1.409	0.961–2.068	0.0795	1.083	0.687–1.708	0.732
PS	0–1	1			1		
	2–3	2.804	1.522–5.165	<0.001	2.243	1.109–4.539	0.0247
Full dose intensity	No	1					
of chemotherapy	Yes	0.924	0.627–1.362	0.69			
Histological diagnosis	DDLPS	1					
	UPS	1.481	0.411–1.495	0.459			
	MFS	1.69	0.841–3.395	0.141			
	LMS	0.783	0.411–1.495	0.459			
	MLPS	0.66	0.269–1.617	0.363			
	MPNST	1.237	0.47–3.254	0.363			
	Others	1.153	0.651–2.043	0.626			
First-line agent	A or AI	1					
	Eribulin	1.199	0.737–1.949	0.465			
	GD	0.89	0.321–2.472	0.824			
	Pazopanib	1.295	0.716–2.343	0.392			
	Trabectedin	1.384	0.627–3.056	0.422			
Retroperitoneal STS	No	1					
	Yes	0.677	0.434–1.055	0.0849			
Best overall response	SD or PD	1			1		
	CR or PR	0.431	0.198–0.939	0.0341	0.537	0.242–1.193	0.127
Target lesion	Both	1			1	1.522–5.165	<0.001
	Local	0.455	0.248–0.835	0.011	0.582	0.296–1.142	0.116
	Metastasis	0.887	0.575–1.371	0.589	0.848	0.518–1.387	0.511

HR: Hazard ratio, 95% CI: 95% confidence interval, PS: Performance status, SD: Stable disease, PD: Progressive disease, CR: Complete response, PR: Partial response, STS: Soft tissue sarcoma. DDLPS: De-differentiated liposarcoma, UPS: Undifferentiated pleomorphic sarcoma, LMS: Leiomyosarcoma, MFS: Myxofibrosarcoma, MLPS: Myxoid liposarcoma, MPNST: Malignant peripheral nerve sheath tumor. A: Doxorubicin, AI: Doxorubicin-ifosfamide, GD: Gemcitabine-docetaxel.

## Data Availability

The datasets generated during and/or analyzed during the current study are available from the corresponding author upon reasonable request.
